# Enzymatic Polymerization of PCL-PEG Co-polymers for Biomedical Applications

**DOI:** 10.3389/fmolb.2019.00109

**Published:** 2019-10-17

**Authors:** Pedro Figueiredo, Beatriz C. Almeida, Alexandra T. P. Carvalho

**Affiliations:** Center for Neuroscience and Cell Biology, Institute for Interdisciplinary Research (IIIUC), University of Coimbra, Coimbra, Portugal

**Keywords:** MD calculations, QM/MM MD simulations, PCL-PEG co-polymers, biodegradable polymers, ROP

## Abstract

Biodegradable polymers, obtained via chemical synthesis, are currently employed in a wide range of biomedical applications. However, enzymatic polymerization is an attractive alternative because it is more sustainable and safer. Many lipases can be employed in ring-opening polymerization (ROP) of biodegradable polymers. Nevertheless, the harsh conditions required in industrial context are not always compatible with their enzymatic activity. In this work, we have studied a thermophilic carboxylesterase and the commonly used Lipase B from *Candida antarctica* (CaLB) for tailored synthesis of amphiphilic polyesters for biomedical applications. We have conducted Molecular Dynamics (MD) and Quantum Mechanics/Molecular Mechanics (QM/MM) MD simulations of the synthesis of Polycaprolactone—Polyethylene Glycol (PCL—PEG) model co-polymers. Our insights about the reaction mechanisms are important for the design of customized enzymes capable to synthesize different polyesters for biomedical applications.

## Introduction

Aliphatic polyesters have attracted great attention in the medical field due to their biodegradability, biocompatibility, and drug permeability, allowing the use of these polymers in biomedical applications (Cameron and Shaver, 2011). However, the hydrophobicity of some of these polymers, such as Polycaprolactone (PCL), still hampers some of their applications (for example, their use as drug delivery vesicles). PCL nanoparticles can be easily absorbed by proteins or be identified and captured by reticuloendothelial cells (Huang et al., 2015). A good way to protect them to be absorbed by proteins, can be achieved by modifying the surface hydrophilicity (Huang et al., 2015). Polyethylene Glycol (PEG), one of the most interesting initiators for synthesis of polyesters, can be used as the hydrophilic part of the linear amphiphilic block co-polymers (Piao et al., 2003; Fairley et al., 2008; Yang et al., 2014). PEG is a non-ionic and water-soluble polymer with biological compatibility, non-toxicity, non-antigenicity, and non-immunogenicity (Panova and Kaplan, 2003). The above mentioned properties, make this hydrophilic polymer widely applied in the pharmaceutical industry and in biomedical applications (Hutanu et al., 2014; Grossen et al., 2017). Recently, it was also employed in the development of polymer-based drug delivery systems. These systems consist in polymers covalently attached to systemic drugs, increasing their molecular weight and thus their circulation time (Hutanu et al., 2014).

Pharmaceutical moieties produced by chemical synthesis, usually contain traces of metals catalysts, which can be a problematic for biomedical application because of their toxicity. Thus, enzymatic synthesis of polymers is considered advantageous and has been extensively studied (Albertsson and Srivastava, 2008; Kobayashi, 2009, 2010; Kobayashi and Makino, 2009; Zhang et al., 2014). Enzymes present many advantages, e.g., they usually operate under mild reaction conditions, they can be highly selective and are biodegradable. The enzymatic synthesis of Polycaprolactone–Polyethylene Glycol (PCL–PEG) triblock co-polymers was reported for the first time in 2003, using Novozyme 435 (immobilized lipase B from *Candida antartica*—CaLB) with fair to good yields (70°C, 63–70% yield), but still with relatively low molecular weights (12.500–17.600 g/mol) (He et al., 2003). A few years later, Huang and his colleagues, used again Novozyme 435 and PEG as the hydrophilic initiator to induce ring-opening of polymerization (ROP) of ε-caprolactone (ε-Cl). They were able to produce amphiphilic co-polymers with slightly higher molecular weights (M_n_ = 11.900–19.000 g/mol at 70°C, 1.28–1.59 polydispersity index). However, these M_n_ values are still low, so approaches with other enzymes or modified enzymes are still required (Huang et al., 2015). Here, in the quest to better understand these processes at atomic level and also to search for alternative enzymes (such as extremophile enzymes, that can withstand harsh industrial conditions), we have studied reaction mechanisms where **PEG** is the initiator in the ROP deacylation step of PCL-PEG co-polymers. We modeled the **PEG** initiator at two different chain sizes. The simpler model consists in a molecule of ethylene glycol and the larger in a polymer with three molecules of ethylene glycol. The initial structure for the Quantum Mechanics/Molecular Mechanics Molecular Dynamics (QM/MM MD) calculations was the second tetrahedral intermediate structure and the simulations were performed with two enzymes: the commonly used CaLB and the thermophilic esterase from the archaeon *Archeoglobus fulgidus* (AfEST).

## Computational Methods

### Systems Initial Setup

The initial structures were modeled from the crystal structures of CaLB (0.91 Å resolution) and AfEST (2.2 Å resolution), pdb codes 5A71 (Stauch et al., 2015), and 1JJI (De Simone et al., 2001), respectively and MolProbity (Chen et al., 2010) was used to assign the protonation states. The enzyme-activated monomer structures (**EAM** with one molecule of ethylene glycol—**MEG** and **EAM** with a oligomer with three molecules of ethylene glycol—**PEG**), the second tetrahedral intermediate structures (**INT-2**) and the product complexes (**PC**, **Co-P** model compound, and **Co-3P** model compound) were geometry optimized in Gaussian09 (Frisch et al., 2009) using B3LYP 6-31G(d) (Ashvar et al., 1996) basis set and with the Polarizable Continuum Model (PCM) (Tomasi et al., 2005) solvent description. The Restrained Electrostatic Potential (RESP) (Bayly et al., 1993) method from HF/6-31G(d) single point energy calculations was used to assign the atomic partial charges. The structures were placed within a pre-equilibrated octahedral box of toluene (10.0 Å between the surface of the protein and the box) and the entire systems neutralized with counter ions. The systems were subjected to two initial energy minimizations and 500 ps of equilibration in a *NVT* ensemble using Langevin dynamics with small restraints on the protein (10.0 kcal/mol) to heat the system from 0 to 300 K. Production simulations were carried out at 300 K in the *NPT* ensemble using also Langevin dynamics with a collision frequency of 1 ps^−1^. Constant pressure periodic boundary conditions were imposed with an average pressure of 1 atm. Isotropic position scaling was used to maintain pressure with a relaxation time of 2 ps. The time step was set to 2 fs. SHAKE constraints were applied to all bonds involving hydrogen atoms (Ryckaert et al., 1977). All the simulations were performed with the Amber molecular dynamics program (AMBER18) (Salomon-Ferrer et al., 2013) using parm99SB (Hornak et al., 2006) and GAFF (Wang et al., 2004) force fields. All reactants, products and intermediate structures were submitted to triplicated simulations of 20 ns each, with different initial velocities. The reference structures represented in the figures, were the lowest root-mean-square deviation (RMSD) structures to the average of the simulations (Dourado et al., 2018).

### Quantum Mechanical/Molecular Mechanical Molecular Dynamics (QM/MM MD) Calculations

The QM/MM MD calculations (Carvalho et al., 2014) were performed using the internal semi-empirical hybrid QM/MM functionality implemented in AMBER18 with periodic boundary conditions. The QM region was described by the PM6 semi-empirical method (Stewart, 2007; Jindal and Warshel, 2016) and the MM region by the Amber parm99SB force field (Hornak et al., 2006). The PM6 Potentials of Mean Force (PMFs) were later corrected with geometry optimizations of the high-level layer (QM) models with the exchange correlation functional basis set for B3LYP/6-31G(d) (Ashvar et al., 1996) and wB97XD/6-31G(d) (Chai and Head-Gordon, 2008), according to Carvalho et al. (2017) and Bowman et al. (2008). Electrostatic embedding (Bakowies and Thiel, 1996) was also employed and the boundary was treated with the link atom approach. Long-range electrostatic interactions were described by an adapted implementation of the Particle Mesh Ewald (PME) method for QM/MM (Nam et al., 2005).

The QM region in the reactant complex for CaLB included: the **MEG** molecule (during the study of **Co-P** production) and the **PEG** molecule (during the study of **Co-3P** production), the S105 residue, the side chain of H224, D187, the amide groups of Q106 and T40, as well as, the side-chain of T40. For AfEST besides the **MEG**/**PEG** molecules and the S160 residue, the QM region also included the side chains of H285, D255, the amide groups of G88, G89, and A161. The initial structure was the **INT-2**, which was obtained using a procedure similar to Escorcia et al. (2017). The reaction coordinate for both enzymes was the distance between the proton of the histidine and the oxygen of the leaving alcohol. The coordinates were scanned in 0.1 Å increments using the umbrella sampling method, except near the transition states were 0.01 Å intervals were applied. The PMFs were computed resorting to the Weighted Histogram Analysis Method (WHAM) (Grossfiled, 2018). The total number of atoms in the high-level layer (QM region) in our initial structure (**INT-2**) was: 77 for CaLB during **Co-P** synthesis and 91 during **Co-3P** synthesis; 67 for AfEST during **Co-P** synthesis and 81 during **Co-3P** synthesis.

## Results

The catalytic cycle of CaLB and AfEST toward the synthesis of PCL through ROP of **ε-Cl** was previously studied (Ma et al., 2009; Elsässer et al., 2013; Ren et al., 2016; Zhao, 2018; Almeida et al., 2019; Pellis et al., 2019) (unpublished data). Both enzymes are able to produce PCL polymers, as already described, and the ability to produce co-polymers of PCL-PEG was outlined in some experimental works (He et al., 2003; Huang et al., 2015), as well as, further explored here, via *in silico* methods. These enzymes have the same catalytic triad, composed by Ser-His-Asp residues (S105-H224-D187 for CaLB and S160-H285-D255 for AfEST) and a hydrogen bond donor region called oxyanion hole, which stabilizes the negative charge developed during the cycle, in the tetrahedral intermediate structure. In these enzymes the histidine residues act as an acid/base (transferring protons between the catalytic serine and the substrate) and are stabilized by the aspartate residue (Brady et al., 1990; Bezborodov and Zagustina, 2014; Douka et al., 2018). The stabilization of the protonated histidine by the aspartate is well-documented (Kobayashi, 2010; Douka et al., 2018). The oxyanion hole region for AfEST contains the backbone amides of G88, G89, and A161 as hydrogen bond donors (De Simone et al., 2001), whereas in CaLB the hydrogen bond donors are the backbone amides of T40 and Q106 and the side-chain hydroxyl group of T40 (Raza et al., 2001).

**Figure 1:**
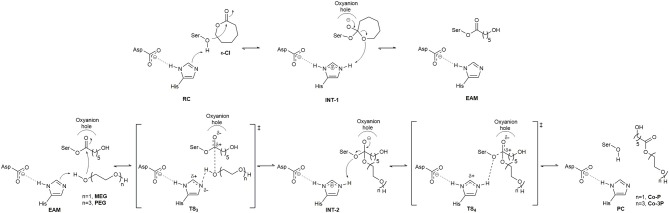
Catalytic mechanism from the formation of the **Co-P** and **Co-3P** products. Above, acylation step (ring-opening of **ε-Cl**); Below, deacylation step.

The catalytic cycle for the synthesis of PCL co-polymers by CaLB and AfEST include the acylation and deacylation steps. The first one (Figure 1), is the nucleophilic attack by the catalytic serine residue to a molecule of **ε-Cl**. This attack leads to the formation of the first tetrahedral intermediate (**INT-1**) structure followed by ring-opening of the **INT-1** resulting in the **EAM** structure. The deacylation steps comprise (Figure 1) a nucleophilic attack to the **EAM** structure by the terminal alcohol function of the initiator (**MEG** when the expected product is **Co-P** and **PEG** when the expected product is **Co-3P**). The result of this attack is the formation of the second tetrahedral intermediate (**INT-2**) structure that, after product release (**Co-P** or **Co-3P**), yields the product complex (**PC**), and the free enzyme is re-generated.

**Figure 2:**
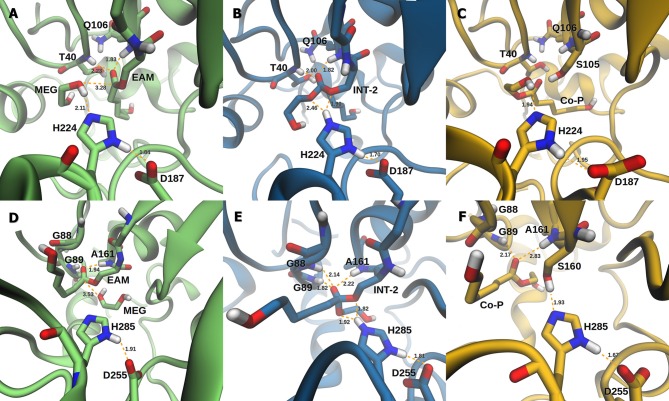
Representation of the **EAM** with **MEG**, **INT-2**, and **RC** with **Co-P** complexes of CaLB **(A–C)** and AfEST **(D–F)**, respectively.

**Figure 3:**
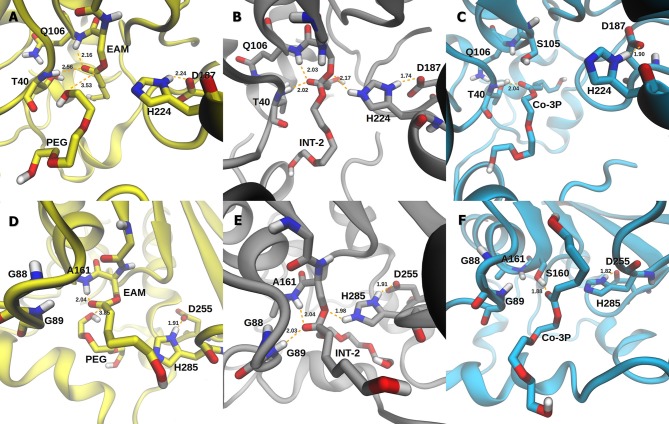
Representation of the **EAM** with **PEG**, **INT-2**, and **RC** with **Co-3P** complexes of CaLB **(A–C)** and AfEST **(D–F)**, respectively.

**Figure 4:**
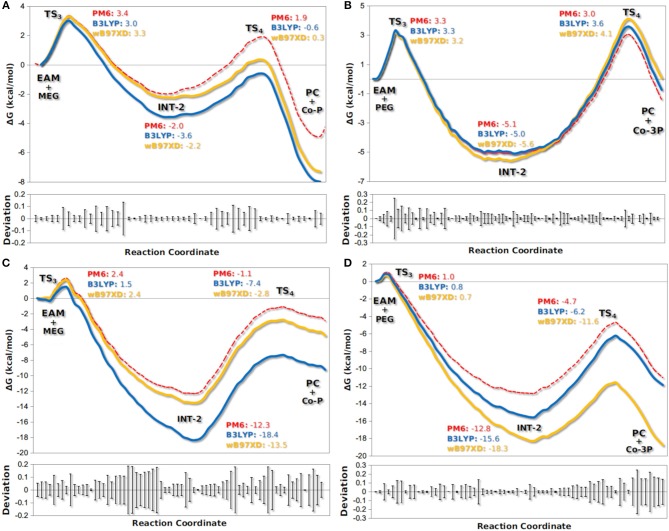
Calculated PMFs for the formation of co-polymers **Co-P (A,C)** and **Co-3P (B,D)** in the deacylation step by CaLB **(A,B)** and AfEST **(C,D)**. Each line denotes the free energies calculated with PM6/MM and corrected with DFT methods (B3LYP with empirical dispersion and wB97XD).

The active site of AfEST is located at the interface of the α/β hydrolase fold with the cap domain, shielding the active site. There is one entrance channel to the active site and two pockets (a large and a medium one, with the latter more buried within the protein) (De Simone et al., 2001). In CaLB there is no cap, just two helices (α10 and α5) that line the active site. The α5 helix was proposed to act as a putative lid (Skjøt et al., 2009; Stauch et al., 2015). The CaLB enzyme pockets have been extensively described in the literature, one binds the acyl moiety (residues A141, L144, V149, D134, T138, and Q157) of the ester and the other one binds the alcohol function (residues W104, L278, A281, A282, and I285) (Wu et al., 2013). The two enzymes have different orientations of the pockets when we compare them (De Simone et al., 2001; Stauch et al., 2015). In AfEST the large pocket has a more hydrophobic nature. Also, since there is just one entrance channel and due to the sequential nature of the events where the **EAM** structure is first formed and then reacts with the initiator, the alcohol function must be located in the larger pocket. We and others have studied the enzyme acylation step in CaLB. The well described rate-limiting step for these enzymatic ROP reactions is usually the formation of the **EAM** structure (Kobayashi, 2010; Huang et al., 2015), excepting with bulky or crowded initiators (Panova and Kaplan, 2003). The acylation step in CaLB, requires around 10.0 kcal/mol (Elsässer et al., 2013) but for AfEST, this barrier is significantly higher (Ma et al., 2009; Li and Li, 2011). As discussed above, the different orientation of the pockets leads to different orientations of the **EAM** structure and hence a different relative position of the attacking alcohol moiety. In the initial **EAM** structures, the incoming alcohol oxygen atom of the initiator (**MEG** or **PEG** molecules) is in a distance range of 3.28–3.53 Å to the **EAM** carbon atom (Figures 2, 3). The reaction proceeds through a transition state (where called **TS_3_,**because of the preceding acylation steps), with concerted proton transfer from the alcohol moiety to the histidine residue and bond forming between the oxygen and the carbon atoms of the **EAM** structure. In the **INT-2** structure, the histidine is well positioned toward the scissile oxygen bond for product formation (the PMFs are represented in Figure 4). In all considered cases, the **TS**_**3**_ barriers are quite low (3.0 ± 0.1 kcal/mol and 3.3 ± 0.3 kcal/mol for CaLB with **MEG** and **PEG** molecules as the initiator, respectively, and 1.5 ± 0.1 kcal/mol and 0.8 ± 0.1 kcal/mol for AfEST with **MEG** and **PEG** molecules as the initiator, respectively—at the B3LYP level of theory correction), which leads to formation of **INT-2** always being exothermic, but significantly higher for AfEST (–3.6 and −5.0 kcal/mol for CaLB; −18.4 and −15.6 kcal/mol for AfEST - B3LYP). The fourth transition state (**TS**_**4**_) free energy barriers are, generally, significantly higher than **TS**_**3**_ for both initiators. With **MEG** molecule as the initiator, the ΔG^‡^ are 3.0 ± 0.1 kcal/mol and 11.0 ± 0.2 kcal/mol in CaLB and AfEST, respectively (B3LYP). On the other hand, when **PEG** molecule is the initiator, the **TS**_**4**_ ΔG^‡^ barriers are 8.6 ± 0.1 kcal/mol in CaLB and 9.4 ± 0.2 kcal/mol in AfEST (B3LYP).

## Discussion

We have studied the nucleophilic attack of **PEG** molecules with different sizes to the **EAM** of CaLB and AfEST enzymes. We found that despite the obvious differences in pockets size, orientation, and lining residues, both enzymes achieve these chemical steps with similar overall energies (with the exception of **MEG** in CaLB that was a lower overall barrier) and that are lower than the barriers in the acylation steps (**TS**_**2**_—Figure 1). In AfEST, the formation of **INT-2** is always more exothermic than CaLB, independently of the substrate. The difference in energies of the **MEG** CaLB reaction in relation to the other reactions seems to be due to the fact that only in this case there is a hydrogen bond between ethylene glycol and the histidine in the reactant complex (**EAM**).

Detailed characterization of the intermediate structures, will allow to identify key residues in the catalytic cycle, opening the door for protein engineering approaches. Enhanced enzyme variants are a good option for industrial esterification reactions (e.g., polyester synthesis) and to improve the biological compatibility of the polymers.

## Data Availability Statement

The datasets generated for this study are available on request to the corresponding author.

## Author Contributions

PF conducted the calculations for the enzyme CaLB and BA on enzyme AfEST. AC supervised the research. All authors contributed to the manuscript writing.

### Conflict of Interest

The authors declare that the research was conducted in the absence of any commercial or financial relationships that could be construed as a potential conflict of interest.
